# The Evolving Family Mealtime: Findings From Focus Group Interviews With Hispanic Mothers

**DOI:** 10.2196/18292

**Published:** 2020-08-10

**Authors:** Amber Jean Hammons, Elizabeth Villegas, Norma Olvera, Kimberly Greder, Barbara Fiese, Margarita Teran-Garcia, The Abriendo Caminos Team

**Affiliations:** 1 California State University Fresno, CA United States; 2 NORC at the University of Chicago Chicago, IL United States; 3 University of Houston Houston, TX United States; 4 Iowa State University Ames, IA United States; 5 University of Illinois at Urbana-Champaign Urbana-Champaign, IL United States; 6 University of Puerto Rico San Juan Puerto Rico; 7 Auburn University Auburn, AL United States

**Keywords:** family mealtimes, healthy eating, technology at the table, parenting and mealtimes, Hispanic culture, obesity prevention

## Abstract

**Background:**

Given the protective effects of shared family mealtimes and the importance of family in the Hispanic culture, this context should be explored further to determine how it can be leveraged and optimized for interventions.

**Objective:**

This study aimed to explore contextual factors associated with family mealtimes in Mexican and Puerto Rican families.

**Methods:**

A total of 63 mothers participated in 13 focus group interviews across 4 states. Thematic analysis was used to analyze transcripts.

**Results:**

Seven overarching themes were identified through the thematic analysis. Themes reflected who was present at the mealtime, what occurs during mealtime, the presence of television, the influence of technology during mealtime, and how mealtimes have changed since the mothers were children.

**Conclusions:**

Hispanic mothers may be adapting family mealtimes to fit their current situations and needs, keeping the television and other devices on during mealtimes, and making additional meals for multiple family members to appease everyone’s tastes. All of these are areas that can be incorporated into existing culturally tailored obesity prevention programs to help families lead healthier lives.

## Introduction

The frequency of shared family meals is associated with healthy eating practices in children and adolescents [1]. Family meals provide an opportunity for family members to connect, and consume healthier food (ie, fewer calories and less fat) than food consumed outside of the home [2,3]. Some studies have also shown a connection between family meals and weight, with the frequency of family meals linked to a reduced risk of being overweight in early adolescent females [4].

Hispanic children have a higher rate of obesity (25.8%) than other ethnic groups [5] and the family mealtime is an important context for promoting healthy eating habits. One study found that 78.3% of Hispanic immigrant families regularly eat a family meal together (ie, every day), and this percentage was even higher when they lived in their home country [6]. Research has revealed that the frequency of shared family meals is associated with increased fiber, fruit, and vegetable consumption in Hispanic youth [7]. Although Hispanic mothers place a high value on family mealtimes, they acknowledge that it is not always possible to regularly eat together as a family [8,9], noting that lack of time and busy schedules present challenges. In addition to shared mealtime frequency, watching television during mealtimes is linked to poorer dietary quality in 10-14-year-old Hispanic children [10]. Given the high value placed on the importance of family in the Hispanic culture, and the protective effects of shared family mealtimes, this context should be explored further to determine how it could be leveraged and optimized for interventions. Thus, the purpose of this study was to explore mealtime frequency and contextual factors associated with family mealtimes in Mexican and Puerto Rican families.

## Methods

### Sample Selection

To participate in the study, participants had to be of Mexican and Puerto Rican descent, and to have a child between 6 and 18 years of age. Participants were recruited via flyers and referrals from community organizations that serve Hispanic families. Flyers were posted and distributed at churches, grocery stores, flea markets, community centers, laundromats, clinics, and recreation centers. Interested families were asked to contact project research staff. A total of 63 mothers participated across 13 focus groups between June 2015 and August 2018. On average, 5 mothers participated in each focus group session, which on average lasted 1 hour in duration. Trained bilingual facilitators conducted the interviews in Spanish at locations that were convenient and comfortable for mothers and ensured privacy (eg, private conference rooms in community clinics, community centers, county extension offices, and university campuses). An interview guide that included the focus group questions and follow-up probes was used for each interview. Previous studies, as well as a review of the literature, informed the development of the guide. The interview questions are included in Textbox 1. Small focus group sessions were selected as the methodology, as the goal was to have open and in-depth discussions with mothers about their family mealtime practices and beliefs.

### Ethics and Consent

This study was approved by the Institutional Review Boards at California State University, Fresno, Iowa State University, University of Houston, and the University of Illinois, Urbana-Champaign. Written informed consent was obtained from all participants prior to conducting the interviews.

**Interview questions.**

Carrying out mealtimes: Mealtimes happen in many different ways for families.

- Who does the cooking and grocery shopping in your home? 
- How often?
- Do your children ever help with either?
- Are your family meals different than when you were a child?

Some families are casual or spontaneous about cooking and shopping. They see what is available and go from here. Others make a plan and use it to shop and cook. And some are in between.

- What is your family like?
- What determines what you serve for meals?
- Do you choose things on sale; use leftovers; have a weekly menu planned ahead?
- What does a “family meal” look like in your house?
- Who is typically there? Describe what all happens during family meals (music, television, conversation, homework, etc)
- How often do you have a “family meal?”
- Are your family meals “traditional”? If so, how or if not, why not?

### Data Collection and Participants

This study is part of a larger multistate randomized controlled trial, *Abriendo Caminos*, an obesity intervention program culturally tailored for Hispanic families [11,12]. The intervention is a 6-week program that focuses on nutrition, family mealtimes, and family physical activity, and is described in detail elsewhere (see [11,12]). Results from this study were used to help guide the content and implementation of the intervention by learning about the needs of the communities in which the intervention would take place. This study was conducted across four states (California, Illinois, Iowa, and Texas) that were selected due to large concentrations of Hispanic families across the state or within rural communities in the state. Focus group interviews, a methodology that is well suited for exploratory research [13,14], were used to explore practices and beliefs regarding family mealtimes among Mexican and Puerto Rican families in each state.

At the beginning of each focus group interview mothers completed a demographic questionnaire that included questions regarding their date and place of birth, generation status, language preference (both spoken and written), educational attainment, religion, marital status, number of children, employment, annual household income, and the number of people the income supports. In addition, a question regarding how mothers rated their general health was included. Mothers were offered an honorarium (ie, US $10 gift card or cash) for their participation in a focus group session.

### Data Analysis

All focus group interviews were audio recorded and transcribed verbatim in Spanish. Then, 2 Spanish proficient, bilingual research assistants independently back-translated the focus groups to English. Next, these back-translated interviews were double-checked for accuracy and phrasing by multiple team members. Dedoose version 8.3.35 [15], a qualitative software analysis program, was used to code transcripts. Thematic analysis, a systematic and iterative approach to help patterns in the data to be discovered and identified, guided the analytical process [16].

Two researchers (AJH and Stephanie Sloane) fully familiarized themselves with the transcripts by reading through them numerous times, and then initially identified codes independently for comparison purposes. Initial code creation was based on ideas that were identified from reading the transcripts as well as preliminary patterns of similarity. Researchers then refined the codes and applied them to the transcripts. As researchers coded the narratives, they continued to compare the codes throughout the process to broaden the comprehensiveness of the codes and ensure consistency in the coding process. In the next step, codes were categorized into initial themes based on the repeated patterns that had been identified in the data. The narratives were then read again to refine the themes. In the final step, quotes were selected that fit these themes [17]. Researchers discussed the appropriateness of the quotes, and a consensus was reached when disagreement was present. Preliminary findings were shared with a note-taker of the focus groups to establish credibility of the findings further and for researchers to enhance the themes.

## Results

### Demographics of Study Mothers

Mothers’ ages ranged between 17 and 74 years (mean 39.8 [SD 10.23]). Most mothers were married (38/63, 60%) and on average had 3 children (mean 3.13 [SD 1.90]). The mean number of years of formal education the mothers achieved in the United States was 4.7 (SD 5.67), compared with 7.96 years (SD 3.54) in their country of origin. More than half (37/63, 60%) of the mothers reported that their families’ annual household income was US $29,999 or less, which is considered a low income for a family of 4 in the United States. The vast majority of mothers (50/63, 79%) were born in Mexico. A small percentage of mothers were born in the United States (8/63, 13%) or other countries not disclosed by mothers (5/63, 8%). Nearly half of the mothers reported that they spoke only Spanish (28/63, 44%) or spoke Spanish better than English (25/63, 40%), and that they read only Spanish (26/63, 41%) or read Spanish better than English (21/63, 33%). On average, the mothers had lived in the United States for 18.5 (SD 7.46) years.

Seven overarching themes, as well as salient quotes to illustrate themes, were identified through thematic analysis. Themes reflected who was present at the mealtime, what occurs during mealtime, the presence of television, the influence of technology during mealtime, and how mealtimes have changed since the mothers were children. Findings are presented across the 4 sites because no consistent regional differences emerged.

### Theme 1: Mothers and Children Are Usually Present at Mealtime

The majority of mothers talked about how their husbands were usually working late, so they (mothers and children) would eat without them.

Yes, only my husband is not present. As long as he brings in the money for food, there is not a problem.

Me too, like normal, never, well, it is very rare that I eat with my husband who comes home around 9 or 10pm “...” only with the children.

My husband eats when he gets a chance. We eat at different times.

Some mothers expanded on this and gave additional explanations for why they would eat separately, including that the children could not wait until father gets home because they were too hungry, and that making them wait would be unfair to them. Therefore, family mealtimes had to occur at different times, and as a result would not include the whole family. For example,

Same, during weekdays is only the baby and me. Same, I make her food, and I eat with her. Moreover, during the afternoon it is normally the four of us because the three girls and my husband gets home very late, he has to eat by himself. My daughters come out of school and get home hungry; it is not fair for them to wait for their father. We do not wait for him because he comes home one hour later. During the weekend, we do have breakfast all together.

A minority of mothers stated they would eat first with their children, and then sit again to eat with their husbands when they got home. In some cases the entire family (with the children), or just themselves and their partner, would be at the table.

My husband does not come home until 6, and sometimes we have already eaten, but when he is at the table, we all sit with him to a eat a fruit or whatever and we accompany him. For me, 6 is too late to eat but my husband does not like to eat alone.

Other mothers said that they also did not want to eat late, because it would be bad for their health, lead to weight gain, indigestion, and/or difficulty sleeping, so they would eat earlier with the children.

### Theme 2: Fathers Are More Likely to Be Present at a Family Meal During the Weekends

Mothers also talked about how fathers would be more likely to share a family meal on the weekends because they usually did not work on the weekends. Fathers’ work schedules, as well as busy schedules, in general, could preclude family meals (where every family member was present) from occurring during the weekdays, but weekends freed up more time.

Almost all family meals are on weekends.

Only on the weekends when we are together, the three of us we eat the three together.

But for job reasons or work schedules, we do not eat together until Saturday and Sunday. Those days we can sit together. However, weekdays it is difficult due to everyone's schedule.

Some mothers shared that the family meal would be a big breakfast, whereas others said it would be lunch or dinner, with some saying all 3 would be meals that would be eaten as a family and specifically reserved for this purpose on the weekends.

### Theme 3: Mealtimes Are for Talking and Checking-in With One Another

Mothers discussed how they liked to spend time during the meals talking and catching up with family members. Some talked about how this is a special time and an opportunity to check-in. They valued this time and viewed it as an opportunity to see how each other’s day went and to see how everyone was doing. Other mothers talked about how it is a way to connect and joke with one another, as one mom shared, “there is chatting going, we tell jokes, and we talk”. Some said the conversations would continue long after the meal ended because family members enjoyed each other’s company and the opportunity to be together.

We talk about how things went at school, how things went at work, because two of my older sons go to school and work too. So, we would talk about what we are doing. I would talk about my work because that is where I let out my stress.

Not all of the family members enjoyed the conversation time in the same way, however, as some mothers talked about how children were eager to get back to television, their phones, or tablets.

At the table we sometimes spend two hours talking and we are not eating anymore; we are just talking. Sometimes my son gets mad because we spend too much time at the table talking. I say, “What is the matter”, he says “I want to use my iPad”. He is young, but sometimes he acts like a big boy, right?

A few mothers talked specifically about rules that they have forbidding devices being at the table, or the television being on, because they want to use this as a time for connection through conversation.

Well since December I have been living alone with my kids. So, right now it is myself and my 3 children at the table. No television or tablets. We use the table time to talk and ask questions like how it went at school, and my children ask me how it went at work. That way, we use the table time for my children to share with me what they think, and what they want.

We usually always eat the three of us together and we do not have the television on. We stopped watching television while eating when I divorced my ex-husband because he did have that habit. But we always try to enjoy the moment and also talk or make plans about what we are going to do in the afternoon, where we are going to go, where we are going to walk, we go to the cinema. I feel that this moment is ours, this moment of eating.

### Theme 4: Television Is Largely a Part of Family Mealtimes

Mothers talked about whether or not the television would be on during the meal. The majority of mothers said the television would be on during the mealtime. For some families, having the television on during mealtime was the norm, but it would sometimes have to be turned off mid-meal because children would be paying more attention to the television than eating. One mother shared,

I do watch the TV. I turn it off if they were not eating (and watching the TV instead). They are only paying attention to the TV and I like to talk to them, ask them what they did and then they talk to me about their adventures.

Some mothers also talked about how family members can be talking and watching television at the same time, “Yes, we have the TV on and sometimes if we do watch TV, we are still talking”, and that that was the norm in their family. One mom stated that “There are times that the TV is on, simply because we are used to talking, we are used to the noise.” A few said that they did not talk during meals, they served their food and then watched television as a family, and that this was preferred over talking with one another*.*

Sometimes I call them so we can eat together but no, it is better to take the food to the living room to eat it in front of the TV.

Each one of us serves our own food and then we go and eat in front of the TV.

In my house, I do not watch TV, but my daughter likes to. If the TV is on, she is not eating well because she likes the movie that is running, she pays attention to it.

Family rules regarding television being on or off was also part of the discussion. Some discussed how they wanted to continue the rules that they had when they were children, while others said the opposite. Some mothers shared how their husbands wanted the television to be on as they looked forward to watching television after a long day after work, and that mothers wanted to respect that, so it would be on during the meal.

In our house, the television is close by. It is actually set in front of us. OK, when my husband arrives, he wants to sit down to eat and watch the television.

While overall, many of the mothers said television would be on during the meal, a few said they would make sure it was off because it was distracting to the children and would prevent them from eating their food. A comment from one mother highlights this,

*No, no I do not have TV or anything like that in the dining room. Because I want the children to focus on the food. And it is healthier.* *So that is the way we manage, we try to focus on talking amongst ourselves. It’s a bit difficult for the children.*

### Theme 5: Influence of Technology on the Family Mealtime

The influence of technology during mealtimes appeared to be mixed. Some of the mothers voiced frustration about devices at the table, saying they would tell the children that they need to eat and to put the devices away. Although some mothers expressed that they did not want technology to be used while eating at mealtime, many of them said that their children insisted on having devices with them during this time, and that it had become a regular source of argument.

Hardly anyone is eating, because they will tell me it is not yummy. And I will tell them to leave cell phones, to put them away so they can focus on their food. And my children tell me “Mama, look at what is happening,” and I will tell them they need to focus on eating!

Some said they felt using technology at the table was disrespectful and that children needed to learn the rules. Others talked about rules they had against having devices present at the table “No television or tablets” and negative emotions around them.

Well, they are married and have their own house, but it makes me angry when we sit down to eat, and they are on their phones.

Well, I punished my son so he can understand me. I told him, “Well, okay, use the cell at the table, but I am going to punish you”. And he did not use it. And he went and left it in his room or where I will not see it.

I try to make sure my daughter does not have her phone or iPod close. She has to do all of her homework and clean her room to have it. It is like a reward.

Still, other mothers said that devices were present during mealtimes and that this was just part of what happens during the mealtime.

Sometimes, there are phones present, or they will go and watch TV.

We will sometimes eat together and at other times, we will eat separately with our cellphones at our sides.

### Theme 6: Mothers’ Approaches to Meeting Multiple Food Preferences

Mothers talked about how they sometimes make multiple meals, one for their children, and sometimes even another separate meal for their husbands, creating a *buffet-style* mealtime. This often results in extra time being spent on meal preparation. Some mothers shared that they will try to get ahead of this problem by only making meals that they know everyone will enjoy, but that this limits the variety in their own diets, as well as in their children’s diets.

That is why I say my house is like a buffet because I ask everyone what they want to eat and then make it. In my house, you will always see different types of food because we all do not eat the same thing; everyone always is eating something different due to their varied tastes. I can eat everything and anything but everyone else does not like eating what others eat.

Sometimes I try for everyone to eat the same thing but normally that does not happen.

Others said they refuse to make multiple meals and children have to eat what is being served, or not eat at all. They commented that they simply do not have the time to cater to each child’s meal preferences.

And now that they spend time with me, they do not eat what I make, because I am not about to make another sort of food. It is double my work ...

That is my husband. He says, “We do not have a buffet, eat what we have or do not eat.”

Nevertheless, many of the mothers said that their children’s preferences are often taken into account when grocery shopping, as a way to ensure that the mealtime will go smoothly.

Yes, I almost always cook something they like to eat because I know they like it. So that way I know what I cooked they will be willing to eat.

In my house, when we go grocery shopping, we always think about what the children will like. If necessary, we will have to make two meals, one for the big ones (adults) and one for the boys, the children.

### Theme 7: Family Meals Are Different From When Mothers Were Kids

Mothers discussed how their mealtimes today are different from what they were like when they were children. Some shared how they would have family meals with everyone present, but today it is harder to do that due to busy schedules, and that when the family sits down together it does not feel *complete.*

Okay, when I was little, the family would have family meals together. Now, we do not eat together as a family because the kids are in school, one goes to work and eats on a different schedule. It is rare when the entire family sits together for a meal.

Well, yes. Here, it is rare when we eat together and back then we would all eat together.

Technology also came up as a large contributor to making today’s family meals different from when they were growing up. They expressed that technology today plays a role in reducing conversation, story time, and physical activity, and there was a sense that their children were missing out on these activities as a result.

I liked it when we were all together, because my husband and I are raising our daughters together but it is a different way of living because now there is the technology and we were raised without technology. We could play all day outside on the street with balls until our parents would holler at us to come home.

When we were in Mexico, it was always the five of us eating together, always.

Now each kid is in their own room or they are using their tablet or watching TV. We do not engage in the same activity as we did. Before, we... my dad and my mom were in the yard. They had us there, my dad would tell stories, everyone around, you know, a bunch of little eyes.

Some mothers also discussed how the types of foods consumed today are different from what they ate when they were children, with a special emphasis on the freshness of the foods they consumed when they were growing up.

## Discussion

### Principal Findings

Knowing about the changing Hispanic family mealtime can help in developing interventions to promote healthy eating habits. According to mothers in the study, family meals, where everyone is present, were more likely to occur on weekends. The use of the television tends to be part of the family mealtime as reported by many mothers. Similarly, phones, tablets, and videos games are all elements of the current societal fabric and mothers take varying approaches to manage their use at the table. Mothers feel that family meals today are different from what they were like when they were growing up. Mothers reported that nowadays there were fewer family meals, where all family members were present. Mothers also indicated that the availability of technology influences meals in ways that did not exist when they were children. Immigrant families face time constraints, including longer working hours, which make juggling work roles and eating regular family meals more challenging [18-20]. Families in this study are still sitting down together to share a meal, just without all family members present. Families are adapting and finding different ways to eat together.

Some mothers discussed that family meals were profoundly meaningful and explicitly reserved for conversation and catching up with one another. While some mothers said that technology was not allowed at the table so as to minimize distractions, others talked about the television being a regular part of the mealtime, which is associated with a reduction in the benefits of sharing a family meal [21,22]. Watching television while eating is associated with a higher caloric intake [3,10] and poorer dietary health in children [22]. One study conducted in Brazil found that having the television on during the meal was associated with a higher intake of chips and parents spending more time encouraging their 6-10-year-old children to eat their food [23]. Another study found that the greater the insistence on eating, the higher the child snack consumption in 3-6-year-olds [24]. This study corroborates these findings as mothers talked about how their children would focus on the television rather than on eating, and they would get frustrated repeatedly asking them to focus on eating. A few mothers also talked about how fathers looked forward to watching television after a long day at work and so the television would be on during mealtimes with fathers. One focus group study found that Hispanic parents believe that turning the television off is an important way to help overweight children lose weight [25]. Getting both mother and father on board with turning the television off during mealtimes should be an important focus of future interventions. Including fun strategies for family time as a substitute for television may be an important component in family-based obesity programs.

In addition to television, other popular technology devices are commonly present at the mealtime and Hispanic mothers are trying to negotiate the use of them during their family mealtime. Some mothers believe strongly that devices should be absent from the table, others seem upset only if the children are distracted to the point of not eating. By contrast, others seem to accept that this is just part of what happens today. Mothers also talked about how their mealtimes when they were growing up were different and that technology presents unique problems today for connection and physical activity. Screen time is something that parents have to navigate today, and while technology carries benefits, finding a healthy balance is important to parents [26]. A mixed-methods study conducted with parents of preschoolers asked parents about their interest in technology use during mealtimes, with a focus on positive purposes such as educating children about healthy eating [27]. Parental interest in technology use during mealtimes was more negative than positive. Parents cited concerns around intrusiveness, reliance on screens, and distraction. While parents are largely uninterested in technology being present at mealtimes, their actual presence at the mealtime seems to be ubiquitous.

With research suggesting that use of technology during mealtimes might be associated with unhealthy eating, interventions should teach parents strategies to minimize the use of technology at the table. Using technology during meals is likely to make connecting with one another, especially through conversation, more challenging, and is also associated with less healthy eating (eg, [28]) and increased risk of overweight and obesity [29]. One family-based intervention, by Fulkerson and colleagues [30] aimed to reduce screen time during meals. Although the intervention was effective at improving child dietary intake, no significant effects were found on the reduction of screen time. However, another family-based intervention, Healthy Habits, Healthy Homes, was effective at reducing child television viewing in general, though the intervention did not target reducing television viewing in the mealtime context specifically [31]. More recently, a family intervention program that focuses on teaching low-income racially diverse parents and children meal preparation skills, Simple Suppers, has been successful at reducing television viewing during mealtimes [32]. Still, more research is needed in this area, especially regarding culturally tailored programs for Hispanic families.

Mothers in this study also reported meal preparation challenges. Because of having children and spouses with picky eating habits, mothers sometimes spent extra time making separate meals for individual family members. Preparing different meals for family members increased their workload and added extra burden to their limited resources. Mothers talked about how they would sometimes plan out meals in advance that they knew their kids would eat, and only purchase and make those foods as a way to minimize workload, conflict, and stress. One study found that making special meals for children with picky eating habits was related to less variety in children’s diets as well as continued refusal to eat the original foods [33]. In a focus group study exploring opinions of intergenerational Hispanic family members, Hispanic youth discussed how parents’ permissive parenting styles around food contributed to children’s unhealthy eating habits [9]. They talked about how parents made it easy for them to develop unhealthy eating habits. Expecting children to eat the same food everyone else in the family is eating may be perceived positively by Hispanic youth when they are older. Sharing these findings with mothers may help empower them to make one meal for all family members, saving both time and energy.

Although fathers were invited to participate in the study, only mothers participated. Thus, the views here represent the experiences and perceptions of mothers only. Nevertheless, mothers are sitting down for mealtimes with their children regularly, even when fathers are not present, and they have an important influence on their children’s dietary habits. Recent research has found that father absence from mealtimes is associated with greater child distractions in 18- to 24-month-old children [34]. Future research may want to examine how Hispanic father absence from weekday mealtimes may influence child behavior during weekend mealtimes. This study also focused only on Mexican, Mexican American, and Puerto Rican mothers. Future studies should examine other Hispanic subgroups to determine whether similar commonalities are found across different Hispanic subgroups. Lastly, there may be situations in which families incorporate certain behaviors, such as television viewing or screen time use, as a means to reduce conflict during mealtimes, relax or get things done, and change the mood [35]. There may be specific intentions behind some of these strategies, and the emotional and mental health of families concerning the use of screen time should be considered in conjunction with their physical health in interventions.

### Conclusions

The Hispanic family meal is evolving, and researchers and practitioners can benefit from knowing what is happening during the meal and what challenges families are facing. The main take-away messages from this study are that Hispanic mothers may be (1) adapting family mealtimes to fit their current situations and needs (2) keeping the television and other devices on during mealtimes, and (3) making additional meals for multiple family members to appease everyone’s tastes. All of these are areas that can be addressed in existing culturally tailored obesity prevention programs to help families create healthier eating habits.
